# The RISE Study: Retrospective Registry for the International Safety and Efficacy Results of Patent Foramen Ovale Closure with Figulla Flex Il PFO and UNI Occluders

**DOI:** 10.3390/jcm13061681

**Published:** 2024-03-14

**Authors:** Nicolas Pioch, Daniela Trabattoni, Helene Bouvaist, Estelle Vautrin, Giovanni Teruzzi, Cecile Dollinger, Gilles Rioufol, François Godart, Alain Fraisse

**Affiliations:** 1 Cardiology Department, University Hospital of Grenoble Alpes, 38700 La Tronche, France; npioch@chu-grenoble.fr (N.P.); hbouvaist@chu-grenoble.fr (H.B.); evautrin@chu-grenoble.fr (E.V.); 2 Centro Cardiologico Monzino, Istituto di Ricerca e Cura a Carattere Scientifico, 20122 Milan, Italy; daniela.trabattoni@ccfm.it (D.T.); giovanni.teruzzi@ccfm.it (G.T.); 3 Fondation Force, 2 rue André Fruchard, 54320 Maxéville, France; rise@fondation-force.fr; 4 Hospices Civils de Lyon, 69002 Lyon, France; gilles.rioufol@univ-lyon1.fr; 5 Centre Hospitalier Universitaire, 59000 Lille, France; f-godart@chru-lille.fr; 6 Royal Brompton & Harefield NHS Foundation Trust, Sydney Street, London SW3 6NP, UK

**Keywords:** patent foramen ovale, interventional catheterization, cryptogenic stroke

## Abstract

**Background:** Transcatheter closure of a patent foramen ovale (PFO) is performed in cryptogenic stroke and other conditions. Information is lacking for some devices. **Methods:** We aimed to evaluate the Figulla Flex II PFO Occluder (FFP) and Figulla Flex UNI Occluder (FFU) through a retrospective multi-center registry. **Results**: 527 patients were included. Mean age was 48.9 (±13.8) years. The procedure was under transthoracic, transesophageal or intracardiac echocardiography in 185 (35.1%), 193 (36.6%) and 149 (28.3%) cases, respectively, and under general anesthesia in 191 patients (36.2%). The FFP and FFU were used in 408 (77.4%) and 119 (22.6%) cases, respectively. The success rate was 99.1%. Median follow-up was 1.1 (0.5–2.5) years. A new atrial fibrillation/flutter within six months occurred in 14 (2.7%) cases, with no difference between devices. One device embolization in the pulmonary artery was identified two years post-procedure. Residual shunts occurred in 18 (6.9%) cases at 1 year, with TIA in three (16.6%) patients. Out of 437 patients with stroke/TIA, 260 (59%) were followed more than one year after closure. Median follow-up was 2.1 (1.17–3.1) years, with four recurrent strokes/TIA. **Conclusions:** The FFP and FFU devices are safe and effective for PFO closure, with very few atrial fibrillation/flutter and neurologic events, except in cases with a residual shunt.

## 1. Introduction

A patent foramen ovale (PFO) exists in around 25% of the population [1]. However, in cryptogenic stroke, a PFO is much more frequent, in approximately 40% of cases [2].

Several recent studies demonstrated that PFO closure is effective in preventing recurrent cryptogenic strokes, between 18 and 60 years of age [2,3,4]. Subsequently, the transcatheter closure of PFO has been approved and multiple devices are currently used for PFO closure [5,6,7,8,9]. However, although PFO closure remains a low-risk procedure, several potentially serious complications have been described [10,11,12], including device migration, erosions, endocarditis and new onset atrial fibrillation or flutter (NOAFF). Long term studies following PFO closure have also demonstrated that there is a small risk of recurrent cerebrovascular events [13,14], possibly related to the type of closure device used [14].

The Figulla Flex PFO Occluder (FFP, Occlutech Holding AG, Schaffhausen, Switzerland) has had CE approval for PFO closure since 2009 (2022 for the last generation Figulla Flex II PFO). The Figulla Flex UNI Occluder (FFU, Occlutech Holding AG, Schaffhausen, Switzerland) is dedicated to treating subjects with a multifenestrated atrial septal defect (ASD), in the presence of clinical symptoms and with a significant left-to-right shunt. However, the FFU has also been used for PFO closure as an off-label device, as an alternative to the FFP, at the initiative of several lead interventionists in various European centers, especially when a large aneurysm of the inter atrial septum (ASA) is present.

Both FFP and FFU are constructed of a super-elastic nitinol wire mesh, polyester patch material and polyethylene terephthalate stitching. The nitinol mesh framework is pre-shaped into two discs that are connected by a thinner waist between them. The left atrial disc is smaller than the right atrial disc for the FFP, whereas both discs have the same diameter for the FFU. Thin polyester patches, placed into each disc, are affixed to the nitinol mesh framework using polyethylene terephthalate sutures to stop blood flow through the defect and to support optimal tissue growth. A ball-shaped connector on the right atrial disc is used to connect the implant to its delivery cable and release mechanism, the Occlutech Pistol Pusher or Flex Pusher I.

Few studies have been published on the safety and efficacy of FFP, mostly including a relatively small number of patients with a short follow-up [9,15,16,17,18], except a recently published single-center study. No study has reported the safety and efficacy of FFU for PFO closure and its mid-term follow-up, with only one case report being available [19].

The aim of the RISE study is to (1) assess safety and efficacy of both FFP and FFU for PFO closure and (2) report the patient’s outcome through a minimal follow-up of 12 months.

## 2. Materials and Methods

### 2.1. Study Design and Eligibility

This is a retrospective, multi-center registry in 4 European tertiary centers performing PFO closure with FFP and FFU. Three were in France (Lyon, Grenoble, Lille) and one in Italy (Milano).

Inclusion criteria was the presence of a PFO requiring device closure before 1 January 2021, for a cryptogenic stroke or any other cause agreed by the investigator: transient ischemic attack (TIA), hypoxemia, decompression illness, intractable migraine, etc. There was no age limit, and any pediatric patients could be included in the registry. Patients with the use of study devices for any condition other than PFO were excluded.

The study steering committee reviewed the data from all subjects to confirm eligibility. The study was approved in France by the heath Authorities «Health Data Hub» and by the local Ethic Committee in Italy. In France, a letter of information and non-objection was sent to every patient, in agreement with the French law for this type of retrospective study. For Italian patients, no consent or information was necessary given the retrospective design of the study. The study was performed in accordance with The Declaration of Helsinki, ICH recommendations and with all applicable laws and regulations of the local country where the study was conducted.

### 2.2. Data Collection/Follow Up

The study coordinators at each participating center collected demographic and clinical data from medical records, using an electronic case report form from the website «clinfile».

Several visits were expected, according to the usual practice regarding a follow-up with reviews at 1, 6 and 12 months, and up to 5 years following PFO closure.

For PFO closure after stroke/TIA, we specifically studied all patients who were followed for more than one year to collect mid-term follow-up data regarding recurrent neurologic episodes.

### 2.3. Objectives

The primary objective was to evaluate the short (up to 12 months) and mid-term (1–5 years) safety and efficacy following the implantation of FFP and FFU by assessing Serious Adverse Device Events (SADEs). SADEs included cardiac erosion, endocarditis, thrombus formation on the device, cardiac tamponade, new onset of atrial fibrillation, or a flutter within 6 months after implantation, and any cardiac or general event that could potentially be attributed to the implanted device or the procedure.

The secondary objectives were a comparison between FFP and FFU, technical and procedural success rate within 24 h post-procedure, PFO closure rate after 6 months and 1 year using bubble test echocardiography, rate of recurrent cryptogenic stroke as well as any other condition that previously represented the indication for PFO closure, the rate and cause of mortality following PFO closure, the frequency and outcome of the new onset of atrial fibrillation or atrial flutter, and the frequency and outcome of other SADEs. In case the indication for PFO closure was migraine, the criteria to fulfill this secondary objective was “totally resolved” or “partially resolved”.

### 2.4. Statistical Analysis

Statistical analyses were performed by methods of commonly applied descriptive statistics including 95% confidence intervals (CIs). The mean ± standard deviation was used for normally distributed continuous variables, the median (interquartile range) for skewed continuous variables and numbers (%) for categorical variables.

Demographics and clinical variables were compared between groups using the Wilcoxon rank-sum test for continuous variables and the Chi2 or Fisher’s exact test for categorical variables, as appropriate.

All statistical results were calculated with Version 9.4 Windows of SAS^®^ software by the French company Statistec (Multihealth Society, Vélizy-Villacoublay, France).

## 3. Results

### 3.1. Patients’ Characteristics and Procedure (Table 1)

**Figure 1:**
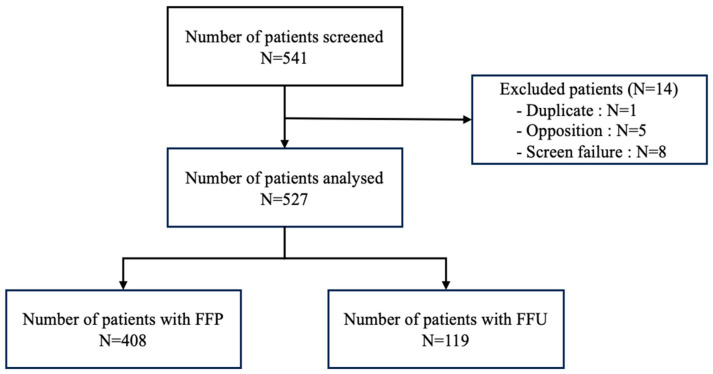
Study profile.

A total of 541 patients who underwent PFO closure between April 2009 and December 2020 were initially included. Fourteen patients were excluded from the analysis (Figure 1), including one duplication, eight screening failures (in four cases, the PFO device was used for another type of defect than a PFO, whereas in four other cases, the date of closure was after 1 January 2021) and five French patients who sent back an objection letter to reject their participation to the study.

**Table 1:** Characteristics of the 527 patients who underwent patent foramen ovale closure with a Figulla Flex PFO Occluder and Figulla Flex Uni Occluder.

Baseline Characteristics	Patient Population—Nb (%) or Mean (± SD)
Female	247 (46.9)
Age (years)	48.9 (±13.8)
Height (cm)	171.2 (±9.3)
Weight (kg)	73.8 (±16.2)
No smoking history	389 (74.0)
Hypertension	134 (25.5)
Hyperlipidemia	120 (22.9)
Diabetes	25 (4.8)
Renal dysfunction	8 (1.5)
Cardiac surgery	20 (3.8)
Coronary angiogram	10 (1.9)
Any other cardiac event	37 (7.0)
Indication for PFO closure	
Stroke	302 (57.3)
TIA	155 (29.4)
Peripheral embolic event	10 (1.9)
Migraine	81 (15.4)
Hypoxemia	21 (4.0)
Decompression illness	5 (0.9)
Professional scuba divers without decompression illness	1 (0.2)
Other	21 (4.0)

TIA, transient ischemic attack.

In the remaining 527 patients, the mean age was 48.9 (+13.8) years and there were 280 males (53.1%). There were several comorbidities, including arterial hypertension in 134 patients (25.5%) and hyperlipidemia in 120 cases (22.9%).

The main indication for PFO closure was a previous stroke (*n* = 302, 57.3%) and TIA (*n* = 155, 29.4%), followed by a migraine (*n* = 81, 15.4%). Seventy-one patients (13.5%) had more than one indication for PFO closure.

The success rate of PFO closure procedure was 99.1%. PFO closure was performed under transthoracic, transesophageal or intracardiac echocardiography in 185 (35.1%), 193 (36.6%) and 149 (28.3%) cases, respectively. General anesthesia was only used for 191 patients (36.2%), including 168 who underwent transesophageal echocardiography monitoring and 23 who had transthoracic echocardiography monitoring. The rest of the patients underwent local anesthesia and/or light sedation. The mean fluoroscopic time was 4.1 (+4.0) min and the mean procedure time was 36.0 (+20.2) min.

During implantation, two (0.4%) procedure-related complications occurred. In one case, the implantation was aborted because of a ST elevation myocardial infarction due to an air embolism during diagnostic catheterization. One patient experienced major bleeding at the puncture site necessitating blood transfusion immediately after the procedure, with a favorable outcome. Additionally, in four patients, the size of the selected device for PFO closure was not appropriate. Another device was then opened and successfully implanted. Finally, three patients experienced mild post-procedure pericardial effusion that was managed conservatively.

### 3.2. SADE and Outcome (Table 2)

All patients attended at least one device implantation visit, including 424 (80%) patients for the 1-month follow-up, 322 (61%) for the 6-month follow-up, and 279 (53%) for the 12-month follow-up visit. A total of 192 (36%) of the patients attended the last follow-up visit when this occurred more than 12 months after PFO closure. Overall, the median follow-up for the entire population was 1.1 (0.5–2.5) years. A total of 507 patients (96.2%) were followed for more than 1 month, 440 (83.4%) more than 6 months and 313 (59.4%) more than 1 year.

**Table 2:** Follow up and serious adverse events after patent foramen ovale closure.

Follow Up	Patient Population (*n* = 527)—Nb (%)
1 month	424 (80.5)
6 months	322 (61.1)
12 months	279 (52.9)
5 years or last follow-up	192 (36.4)
SADEs following implantation.	
Cardiac erosion	0 (0)
Endocarditis	0 (0)
Thrombus formation on the device	0 (0)
Cardiac tamponade	0 (0)
NOAFF within 6 months after implantation	14 (2.7)
Device embolization	1 (0.2)
Neuro events after PFO closure for stroke/TIA	Population >1 y F/U (*n* = 260)—Nb (%)
Recurrent TIA	2 (0.76)
Recurrent stroke	2 (0.76)

Neuro, neurologic; NOAFF, new onset of atrial fibrillation or flutter; SADE, severe adverse device event; TIA, transient ischemic attack; y, year.

Fifteen patients (2.8%) experienced at least one SADE within 1 year after PFO closure, including the new onset of supraventricular arrhythmia in 14 (2.7%) cases.

**Figure 2:**
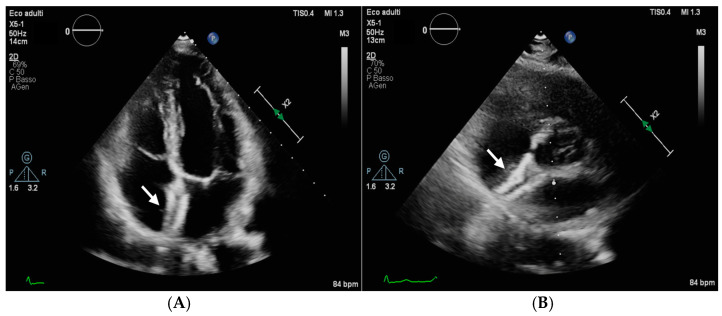
Six-month follow-up transthoracic echography of the patient who experienced late device embolization showing a well-positioned FFP device in a four-chamber view ((**A**), arrow) and in a parasternal short-axis view ((**B**), arrow).

One device embolization was diagnosed 2 years after implantation in a 45-year-old patient who underwent PFO closure for a migraine with a 27/30 FFP device. The 6 month follow-up transthoracic echocardiography demonstrated a good position of the device (Figure 2). The patient did not have symptoms and the embolized device was found to be in the proximal left pulmonary artery. Because this was not causing a significant obstruction of pulmonary blood flow, it was decided to leave the device there. The residual ASD was sized at 2 cm and subsequently closed with a 22 mm Amplatzer Septal Occluder (Abbott, Chicago, IL, USA) device.

Residual shunts at 12 months were observed in 18 (6.9%) out of 261 patients who underwent a bubble test echocardiography. In all of them, the indication for PFO closure was a stroke or TIA. A total of 3 (16.6%) out of the 18 patients with a residual shunt experienced recurrent neurologic events with TIA in all of them.

Out of 437 patients who underwent PFO closure because of stroke or TIA, 260 (59%) were followed for more than one year. The median follow-up for this sub-group of 260 patients was 2.1 (1.17–3.1) years. Four (1.54%) experienced recurrent strokes (*n*= 2) or TIA (*n*= 2) during the follow-up period.

Interestingly, only 12 patients out of 424 (2.8%) reported the new onset of a migraine at the 1-month follow-up visit.

### 3.3. Comparison of FFP with FFU Devices

PFO closure was predominantly performed with FFP in 408 patients (77%), whereas 119 underwent closure with FFU (23%). There were no significant differences between the FFP and FFU population (Table 3). Regarding the indication for PFO closure, patients with TIA (*n* = 133—32.6%) and migraine (*n* = 73—17.9%) were more frequent in the FFP population, whereas PFO closure for other causes were more frequently encountered in the FFU patients.

During the procedure, local anesthesia was more frequently used for FFU implantation (*n* = 90, 75.6%), with a longer procedural time than with FFP: 46.2 (+21.3) vs. 32.1 (+18.4) min. Echocardiography guidance for implantation was more frequently TEE (*n* = 167—40.9%) and ICE (*n* = 136—33.3%) for FFP implantation and TTE (*n* = 80—67.2%) for FFU implantation.

The rate of new onset atrial fibrillation of atrial flutter was similar between the two devices, as well as residual shunts.

## 4. Discussion

With more than 500 patients undergoing PFO closure, this multi-center study is the largest performed with the Occlutech devices. The results support the safety and efficacy of the FFP and FFU, with a low rate of reported complications. Importantly, this is the first study to describe PFO closure with the FFU, used as an off-label device, in a large population. The available procedural and follow-up data show excellent results and outcome. Finally, for 260 patients followed more than one year after PFO closure for stroke/TIA, there were very few (1.54%) recurrent strokes/TIA.

Our study reports mid-term follow-up in patients undergoing PFO closure. This provides important information, especially regarding the percentage of residual shunts as well as the risk of recurrent neurologic events. Most of the previous studies have reported the safety and efficacy of the FFP in a small patient population, and with a shorter follow-up. However, Snijder et al. reported a follow-up of 5.9 ± 1.8 years in 250 patients following PFO closure with FFP [9]. More recently, Trabattoni et al. analyzed 446 patients in their study with a maximal follow-up of 10 years [20].

Not surprisingly, the most frequent indication for PFO closure with FFP or FFU is cryptogenic stroke. However, an important patient population with only TIA was selected for PFO closure. Although studies are lacking on PFO closure after TIA, there is growing evidence that such patients may benefit from this procedure. A recently published study comparing PFO closure after stroke or TIA found similar characteristics and outcome in both populations [21]. Another growing indication for PFO closure in this study is a migraine. Although this is still controversial, recent studies have emphasized the potential benefit of PFO closure in this patient population, especially in severe migraines with aura [22,23].

With less than 7% of residual shunts at 12 months with both FFP and FFU, our study compares favorably to other studies performed with different types of devices, reporting between 4.5% and 34% of residual shunts [2,3,5,24]. However, with almost 17% of TIA in the 18 patients with residual shunts one year after PFO closure, the risk of a recurrent neurologic event seems important in this group of patients. Previous studies also demonstrated such increased neurologic risk with identification of several risk factors including ASA, moderate or severe residual shunts [25]. Most of those residual shunts can be successfully closed during another transcatheter procedure [26].

The present study is the first to report the routine use of the FFU for PFO closure. Previously, only one case report was published about successful PFO closure with the FFU [19]. The FFU was initially designed for multifenestrated ASD closure. However, the main differences between the FFU and FFP devices are the similar diameter of both the proximal and distal discs in the FFU and a larger maximal diameter than the FFP. Some other PFO devices have, nowadays, a similar design than the FFU, like the Cocoon PFO Occluder (Vascular Innovations Co., Nonthaburi, Thailand) [8]. Additionally, PFO closure has been successfully reported with the Amplatzer Cribriform device (Abbott, Chicago, IL, USA), a device with a similar design that is also dedicated to multifenestrated ASD closure [27,28].

Interestingly, TTE was more frequently used when PFO was closed with a FFU device. The longer fluoroscopy time with the FFU is possibly related to the increased use of TTE guidance because TEE and ICE can probably be used more efficiently to guide PFO closure without fluoroscopy [29,30,31]. Additionally, because the FFU device seems increasingly used in complex PFO with large ASA, one could speculate that more patients who underwent PFO closure with the FFU underwent a pre-procedure TEE to fully assess the morphology of the defect. In such cases, the type of device is selected from the pre-procedure TEE and a transcatheter closure can subsequently be performed under TTE. Interestingly, even if the FFU was maybe used in more complex PFO morphologies than the FFP, the risk of recurrent neurologic events was not significantly different between both devices.

In the present study, we found one very unusual case of late embolization with a FFP that migrated to the left pulmonary artery. Device embolization after PFO closure is extremely rare and mostly occurs during or shortly after the transcatheter procedure. However, late embolization has been previously reported, although the mechanism for this remains unclear [32]. In our case, the embolization occurred between 6 months and 2 years after the procedure. Possibly, the initial size defect was underestimated and a 22 mm Amplatzer Septal Occluder was necessary to close the residual defect after the embolization was diagnosed. The initial procedure was performed under ICE guidance. However, a recent study found significant differences between ICE, TEE and the measurement of the PFO with a sizing balloon, concluding that imaging methods may sometimes dramatically underestimate the size of the defect [33].

In our patient population, the rate of NOAFF was 2.7%. This is similar to other studies [34,35]. Importantly, the decision to only include, among SADEs, the NOAFF occurring within 6 months following PFO closure was based on the results of several studies, including a meta-analysis, showing that the vast majority of those post-procedure arrhythmias occur during this 6-month period [34,35].

In the present study, there were no reports of serious nickel hypersensitivity. Moreover, less than 3% of patients reported a migraine at the one-month follow-up visit. Although these symptoms might be under-reported in cases with a mild degree of migraine, it is interesting to note that the Occlutech devices are made of Titanium oxide-covered nitinol, which should result in the lowest possible release of nickel [36]. Nickel allergies are type IV: contact allergies caused by sustained exposure, usually after several hours or days. Allergy to titanium is also reported, with a much lower incidence. Closure with nitinol-containing prosthetic cardiac implants is not contraindicated in patients with nickel allergy because 8% to 19% of the population has this [37]. Most patients will not experience any symptoms after device closure. However, intravascular exposure to nickel can exceptionally cause systemic reactions, due to nickel elution. Nickel hypersensitivity manifests with varied clinical symptoms, especially migraine headaches, but also palpitations, shortness of breath, chest discomfort, back pain, urticarial, rash, pericardial effusion and angioedema [38]. Few extreme cases happened several years after the implantation of devices, possibly due to incomplete endothelialization. Patients underwent surgical explantation, with the subsequent resolution of symptoms [39].

This study has several limitations, including the retrospective design of the registry. Additionally, the participating centers were all tertiary and experienced centers for PFO closure, which may have provided better results than a registry including less experienced centers. Moreover, because many patients were subsequently followed in local hospitals, some SADEs and outcome data may have been under-reported. The existence of 155 patients who underwent PFO closure following TIA may complicate the interpretation of the study as TIA is not an approved indication for PFO closure. However, this also reflects the current “real life” practice in many European centers. Although we showed that FFU is as safe and effective as FFP, our study failed to clarify the specific indications for PFO closure with the FFU. We can only speculate that aneurysmal PFO is generally an appropriate indication. Similarly, we do not know the rate of interatrial septal aneurysm in patients with a residual shunt and recurrent neurologic event. Further comparative studies should include a detailed description of the defects to better determine which PFOs are more suitable for FFU.

## 5. Conclusions

Our data support that the FFP and FFU are both safe and effective for PFO closure, with very few recurrent neurologic events and a low rate of new onset atrial fibrillation or flutter during follow-up. More studies are needed with longer follow-up and accurate descriptions of the morphology of the PFO, especially to clarify the specific indications of the FFU for PFO closure. In addition, more randomized trials are necessary to confirm the effectiveness of PFO in TIA and migraine.

## Figures and Tables

**Table 3:** Comparison between the Figulla Flex PFO Occluder and Figulla Flex Uni Occluder.

Patients’ Characteristics	FFP (*n* = 408) Nb (%) or Mean (±SD)	FFU (*n* = 119) Nb (%) or Mean (±SD)	*p*-Value
Female	191 (46.8)	56 (47.1)	0.96
Age (years)	48.2 (+13.2)	51.5 (+15.3)	0.03
Height (cm)	171.1 (+9.2)	171.6 (+9.8)	0.47
Weight (kg)	72.8 (+15.3)	77.1 (+18.7)	0.05
No smoking history	309 (75.9)	80 (67.2)	0.04
Hypertension	98 (24.0)	36 (30.8)	0.14
Hyperlipidemia	92 (22.5)	28 (23.9)	0.75
Diabetes	15 (3.7)	10 (8.5)	0.03
Renal dysfunction	6 (1.5)	2 (1.7)	1.00
Other cardiac surgery	13 (3.2)	7 (6.0)	0.17
Other cardiac events	25 (6.1)	12 (10.3)	0.12
**Indication for PFO closure**			
Stroke	227 (55.6)	75 (63.0)	0.15
TIA	133 (32.6)	22 (18.5)	0.003
Peripheral embolic event	6 (1.5)	4 (3.4)	0.24
Migraine	73 (17.9)	8 (6.7)	0.003
Hypoxemia	14 (3.4)	7 (5.9)	0.28
Decompression illness	2 (0.5)	3 (2.5)	0.08
Professional scuba divers without decompression illness	0 (0)	1 (0.8)	0.23
Other	7 (1.7)	14 (11.8)	<0.001
**Procedural data**			
General anesthesia	162 (39.7)	29 (24.4)	0.002
Fluoroscopy time (min)	3.9 (+4.1)	4.6 (+3.7)	0.002
Procedural time (min)	32.1 (+18.4)	46.2 (+21.3)	<0.001
TTE	105 (25.7)	80 (67.2)	<0.001
TEE	167 (40.9)	26 (21.8)	<0.001
ICE	136 (33.3)	13 (10.9)	<0.001

FFP, Figulla flex PFO Occluder; FFU, Figulla flex Uni Occluder; ICE, intracardiac echocardiography; TEE, transesophageal echocardiography; TIA, transient ischemic attack; TTE, transthoracic echocardiography.

## Data Availability

The data presented in this study are available upon request from the corresponding author. The data are not publicly available due to privacy.
